# A Rare Case of Solitary Fibrosus Tumor (SFT) of the Nasal Septum

**DOI:** 10.22038/IJORL.2023.72285.3450

**Published:** 2023-11

**Authors:** Davide Burrascano, Salvatore Dolores, Angelo Immordino, Francesco Lorusso, Francesco Dispenza, Federico Sireci

**Affiliations:** 1 *Department of Biomedicine, Neuroscience and Advanced Diagnostics, AOUP Paolo Giaccone, University of Palermo, Via del Vespro, 133, 90127 Palermo, Italy.*

**Keywords:** Solitary fibrosus tumor, nasal tumors, differential diagnosis, nasal surgery, plasma blade

## Abstract

**Introduction:**

Solitary fibrous tumor (SFT) is a rare mesenchymal tumor that usually arises from the pleura but can also occur in extrapleural sites, such as the sinonasal region. It causes aspecific symptoms, including nasal obstruction and discharge, postnasal drip, anosmia, epistaxis, and headache. It may be difficult to distinguish these symptoms from those caused by other mesenchymal lesions that usually occur in this site, especially when the tissues undergo iatrogenic damage following surgical removal.

**Case Report:**

This case report shows a rare right nasal septal solitary fibrous tumor, which was surgically removed using a trans-nasal endoscopic technique. For the first time, the mass was decomposed by a plasma blade, and the implant site was treated by performing a subperiosteal removal of septal mucosa and cartilage. Histopathological examination confirmed the diagnosis of solitary fibrous tumor. Follow-up at three, six, and twelve months showed no signs of relapse.

**Conclusions:**

Sinonasal SFT is unusual, and it may be difficult to distinguish it from other mesenchymal lesions in this site. In the literature, cases treated with CO_2_ laser are usually described; however, due to the high cutting temperatures, this can cause thermal damage of the tissues, making histopathological diagnosis difficult. The plasma blade uses pulsed radiofrequency, creating an effective cutting edge while the blade stays near body temperature. Therefore, this device results in atraumatic, scalpel-like cutting sensitivity and electrosurgical-like hemostasis, with minimal bleeding and tissue injury. Its use could, therefore, help both the surgeon in obtaining surgical radicality and the pathologist in the correct histologic classification.

## Introduction

Extrapleural solitary fibrous tumors (SFTs) are rare submesothelial fibroblast-like mesenchymal tumors that typically arise from the parietal or visceral pleura and exhibit intermediate biological behavior. They may also develop in extrapleural sites of mesenchymal origin, including the peritoneum, mediastinum, lung, nose and paranasal sinuses, larynx and parapharyngeal space, intra- and epidural spaces, orbit, thyroid, and upper airways. 

SFTs have low sex predilection (M:F=5:4) and present clinically in the 5th-6th decades of life with aspecific symptoms or symptoms related to space-occupying masses. Sinonasal SFTs usually arise from the lateral wall of the nasal fossa and ethmoid sinus, and patients show most frequently with nasal obstruction. Other symptoms may include nasal discharge, epistaxis, anosmia, postnasal drip and headache (1). 

Although the etiology remains unknown, recent evidence suggests that a NAB2-STAT6 gene fusion due to a paracentric inversion on chromosome 12q13 is involved in SFTs’ pathogenesis. The NAB2-STAT6 gene encodes a chimeric protein that activates ERG1 and appears to deregulate ERG1-dependent target genes (2,3). 

SFTs share a similar growth pattern with haemangiopericytomas (HPCs) and can be classified as HPC-like neoplasms. Three groups of HPC-like neoplasms can be identified: non-HPC neoplasms, which occasionally display HPC-like features (e.g., synovial sarcoma); lesions with clear evidence of myoid/pericytic differentiation (true HPCs); and fibrous-to-cellular SFTs and related lesions such as giant cell angiofibromas and lipomatous HPCs (4).

Immunohistochemical expression of STAT6 can support in the diagnosis and differentiation of SFTs from other spindle cell lesions in this site. However, sinonasal SFTs are unusual and can be difficult to distinguish from other mesenchymal lesions, especially when the tissues undergo iatrogenic damage following surgical removal (5,6). 

This case report shows a rare right nasal septal solitary fibrous tumor which was surgically removed by using for the first time a plasma blade through a transnasal endoscopic technique. 

## Case Report

A 60-year-old male patient presented to our ENT unit with a complaint of a 30-year-history of right-sided nasal obstruction, anosmia, rhinorrhoea, postnasal drip, and frontal headache, as well as bilateral epiphora for 5 years. He had no history of nasal bleeding or vision disorders. His medical history included GERD, systemic arterial hypertension, left leg deep vein thrombosis, migraine, osteoporosis, and skin melanoma. Nasal endoscopy revealed a large, lobulated tumor with a hard consistency that originated from the nasal septum of the right nasal cavity and extended from the nasal vestibule to the ipsilateral choana. 

**Fig 1:**
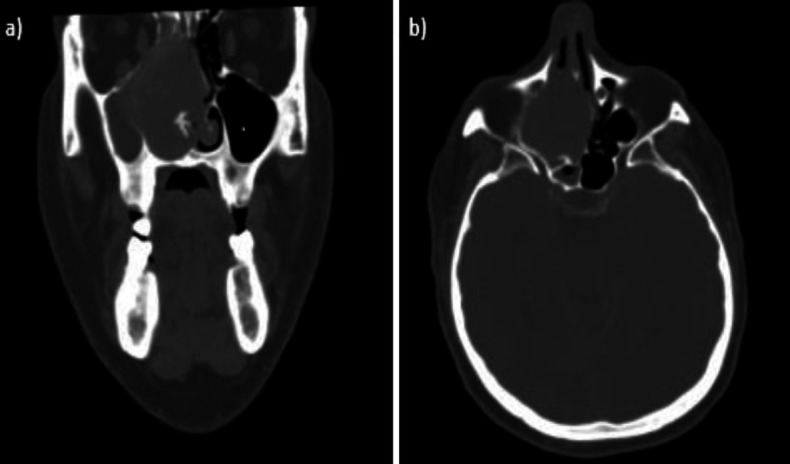
a) CT image of the SFT in coronal section, b) CT image of the SFT in axial section

Computed Tomography (CT) scans showed an oval mass with lobulated margins, parenchymatous density, which displaced the nasal septum to the left, the right nasal wall to the right, the right ethmoid cells superiorly, the unciform process, and the right lamina papyracea superoexternally, which it was in continuity with (Fig. 1). 

**Fig 2:**
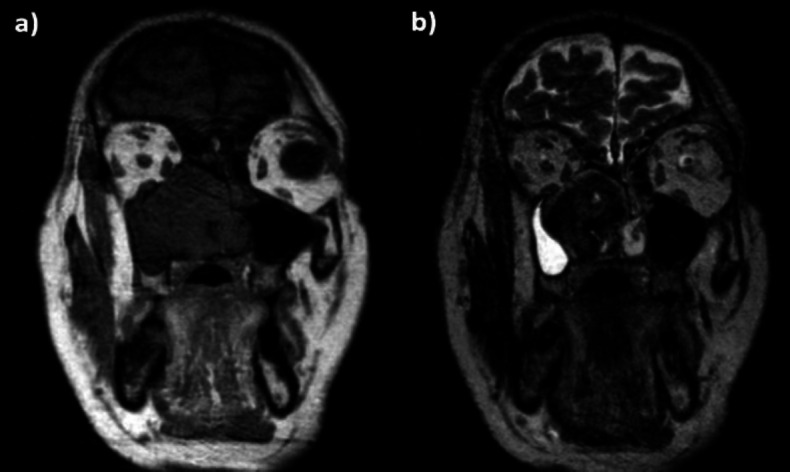
a) T1 weighted MRI, b) T2 weighted MRI

On Magnetic Resonance Imaging (MRI), the tumor appeared uneven intensity, mainly medium-low with hypointense and hyperintense components in T1, mainly low with hyperintense components in T2 and STIR (Fig. 2a and 2b). 

A preoperative biopsy was performed under local anesthesia, and the histological examinations correlated the mass with an antrochoanal polyp. 

**Fig 3:**
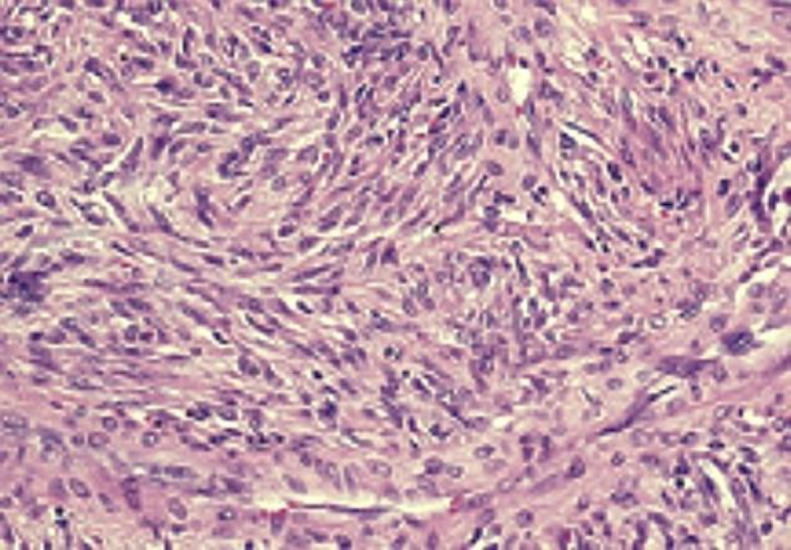
microscopic features of the tumor (100x)

The patient underwent general anesthesia for transnasal endoscopic removal of the tumor. The attachment was suspected to be in the septal region, and therefore, we started disassembling the tumor by using a plasma blade. The insertion was identified and a subperiosteal removal of the septal mucosa was performed. An additional adherence area was found posteroinferiorly at the level of the choanal border, which was eliminated by bipolar forceps. During the surgical procedure, it was necessary to cauterize the septal branch of the sphenopalatine artery (7). Finally, uncinectomy and media antrostomy were performed, and a nasal packing was placed in the nasal cavity. The packing was removed two days later (8). The final histology examination, performed by two different pathologists, revealed a homogeneous proliferation of spindle cells that alternated between hypercellular and less cellular areas with collagenized stroma, characterized by occasional mitoses (Figure 3). 

**Fig 4:**
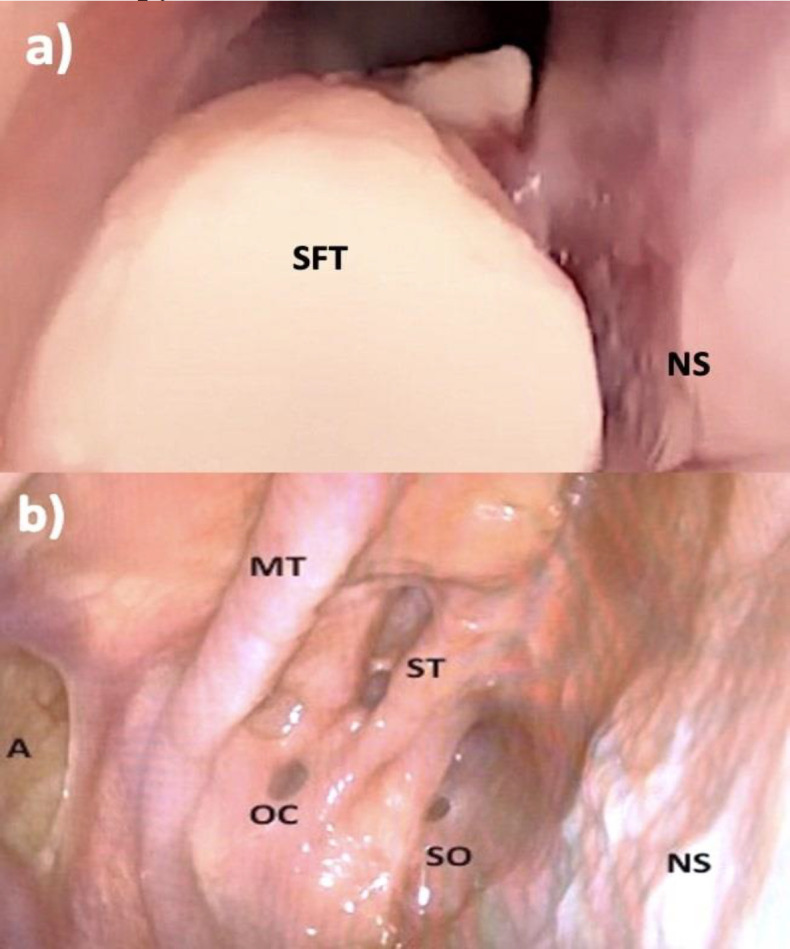
a) preoperative nasal endoscopy: SFT entirely occupying the right nasal fossa. SFT, Solitary fibrous tumor; NS, Nasal septum. b) Two years postoperative nasal endoscopy: absence of recurrence in the nasal nostril. A, Antrostomia; MT, Middle Turbinate; OC, Onodi Cell; Superior Turbinate; SO, Sphenoid Ostium; NS,  Nasal Septum

The immunohistochemistry testing revealed that neoplastic cells exhibited positivity for vimentin, CD34, and CD99. The diagnosis of SFT was confirmed, and examination of the margin revealed complete removal of the tumor. The patient underwent follow-up examinations at 3, 6, 12, and 24 months and remained free of disease. The patient was discharged and it was recommended to perform saline nasal irrigation. After oncology counselling, adjuvant radiotherapy or chemotherapy was not performed because the margins were free and the histotype had an indolent course. Figure 4 shows preoperative (a) and two years postoperative (b) nasal endoscopy.

## Discussion

Benign tumors of the sinonasal tract can be categorized into several groups depending on their histological nature, including fibro-osseous, neural, hamartomatous, odontogenic, vascular, and inverted papilloma (9). Malignant tumors account for only 35% of all head and neck neoplasms (10-13). The most common sinonasal malignancies are primary epithelial malignancies, followed by non-epithelial malignancies (14,15). 

However, extrapleural SFTs belong to tumors with borderline behavior and low malignant potential of soft tissue. SFTs are submesothelial, fibroblast-like mesenchymal tumors that typically develop in the parietal or visceral pleura. However, they can also be found in extrapleural sites such as the peritoneum, mediastinum, lung, nose and paranasal sinuses, epiglottis and parapharyngeal space, intra- and epidural space, orbit, thyroid, and upper airway. 

The incidence is higher in the 5th-6th decade of life, and the most affected sex is male. Symptoms are usually aspecific or related to the space-occupying mass and most often lead to unilateral nasal obstruction. MRI shows a hypo- or isointense mass at T1 weighing and hypo- or hyperintense at T2 weighing, with heterogeneous enhancement after contrast (16).

Immunohistochemically, SFTs are characterized by CD34 expression. According to WHO, the criteria for defining a malignant case, include hypercellularity, increased mitoses (>4 mitoses per 10 high-power fields), cytologic atypia, tumor necrosis, and/or infiltrating margins (5). The most important prognostic factor is the resectability of the lesion (17,18). Only nine cases of SFT in the sinonasal tract have been described in the literature. If we consider only those with an implantation base at the level of the nasal septum, the number of cases drastically reduces (Table 1).

**Table 1:** Sinonasal SFT cases in literature, their implantation site and surgical approach

**Authors**	**Year**	**Age**	**Sex**	**Site of implantation**	**Surgical Approch**
Cavaliere et al. (16)	2020	55	♂	Anteriorsuperior region of the nasal septum.	Removed the tumor, achieving mucosal flap with submuchoperichondrium incision.
Rizzo et al. (19)	2015	26	♂	Not specified.	Embolization; endoscopic piecemeal resection of the mass; resection of the periosteum of the bones that the tumor contacted.
Fujikura et al. (20)	2012	37	♀	Superior turbinate.	Endoscopic resection of the superior turbinate.
Janjua et al. (21)	2011	33 36 41	♀ ♂ ♂	Not specified.	Endoscopic resection.
Eloy et al. (22).	2006	26	♀	Not specified.	Endoscopic resection with opening of the air cell compartments of right ethmoid sinus and middle turbinectomy.
Hicks et al. (23)	2004	31	♀	Dura of the anterior cranial fossa.	Transcranial approach, with bicoronal skin flap and subfrontal extradural exposure
Konstantinidis et al. (24)	2003	47	♀	Caudal end of the left inferior turbinate.	(Local anesthesia) Excised en block with the caudal end of the inferior turbinate

In all the cases described in the literature, the patients complained of unilateral nasal obstruction. The diagnostic procedure included endoscopic examination, imaging tests (CT and/or MRI), and preoperative biopsy. 

The surgical approach was a complete endoscopic transnasal excision, except in one case (Rizzo et al.) where it was preceded by embolization due to the high vascularity of the mass (19).

In our case, the patient had benign histological criteria (i.e. hypocellularity, low number of mitoses - <4 mitoses per 10 high-power fields -absence of cytologic atypia, tumor necrosis, and/or infiltrating margins), and therefore, surgery without chemo-radiotherapy was the treatment of choice. Figure 3 shows the microscopic features of the tumor. We performed an endoscopic resection of the mass with excision of its attachment to the mucoperichondrium with cartilage. However, an accurate differential diagnosis between SFT and a metastasis/recurrence of melanoma was required because the patient had a history of skin melanoma. 

In fact, especially in metastases, melanoma can lose melanocyte lineage-specific markers, presenting with an unusual morphology and immunohistochemical characteristics similar to those typical of SFTs.

Bekers et al. reported two cases of SFT-like melanoma in two women with distant metastasis several years after primary surgery. In both cases, the metastasis/recurrence showed positivity to CD99 and CD34, negativity to typical melanocyte markers (S-100, HMB-45, MART, Tyrosinase, and SOX-10), and morphological differences from the primary tumor. In these situations, the most useful tool is PCR study of mutations for BRAF, NRAS, KIT, GNAQ, and GNA11 that confirm the diagnosis of recurrent/metastatic melanoma (25). The study by Sireci et al. retrospectively analyzed the database of two University Hospitals (Genova and Palermo) on the type of treatment given to sinonasal tumors from 2012 to 2020. A cohort of 32 patients with tumors of the nasal septum was detected. A total of 28 (87.5%) cases were benign neoplasms, and four (12.5%) cases were malignant tumors. The study showed that in cases of benign tumors of epithelium or stroma, the surgical approach chosen was resection of mucoperichondrium/ periostium. In all malignant tumors, resection of all layers of the nasal septum was performed. In the case of relapsing benign tumors with malignant foci, and with underlying hyperostosis, like our case of SFT, mucoperichondrium/ periostium with cartilage/ bone was resected (26).

In the case described, we preferred the endoscopic transnasal approach and the "disassembling technique," which consists of the anatomical-oriented decomposition of the mass to find the exact point of origin (27).

## Conclusion

The literature describes cases in which the tumor mass resection was performed by using a CO_2_ laser fiber. However, this procedure causes high thermal energy with possible damage of the tissue, such as shrinkage and carbonization of the margins leading to difficult pathological examination. In our case, for the first time, we used a plasma blade for the decomposition of the mass. The plasma blade uses pulsed radiofrequency to generate a plasma-mediated discharge along the exposed rim of an insulated blade, creating an effective cutting edge while the blade stays near body temperature therefore, this device results in atraumatic, scalpel-like cutting sensibility and electrosurgical-like hemostasis, with minimal bleeding and tissue injury.The presence of SFT in the sinonasal region is a rare occurrence and can cause aspecific symptoms. Due to its intermediate behavior and unpredictability, an extended surgical approach is necessary, using the disassembling technique to identify the implantation site of the mass. It is also crucial to differentiate SFTs from skin melanoma metastasis, especially in patients with a history of this skin lesion. For tumors with an implant base on the nasal septum, the extent of resection is critical, with a multilayer approach for malignant cases and a mucoperichondrium/ periostium with cartilage/ bone in benign cases with malignant foci. In these cases, the plasma blade could be a viable alternative to laser CO_2_ fiber, providing effective cutting with minimal tissue damage and helping both the surgeon in obtaining surgical radicality and the pathologist in the correct histologic classification.
